# Transfusion-Transmitted Zika Virus Infection in Pregnant Mice Leads to Broad Tissue Tropism With Severe Placental Damage and Fetal Demise

**DOI:** 10.3389/fmicb.2019.00029

**Published:** 2019-01-23

**Authors:** Wanbo Tai, Denis Voronin, Jiawei Chen, Weili Bao, Debra A. Kessler, Beth Shaz, Shibo Jiang, Karina Yazdanbakhsh, Lanying Du

**Affiliations:** ^1^Lindsley F. Kimball Research Institute, New York Blood Center, New York, NY, United States; ^2^Key Laboratory of Medical Molecular Virology of MOE/MOH, Fudan University, Shanghai, China

**Keywords:** Zika virus, blood transmissibility, broad tissue tropism, placental infection, fetal demise

## Abstract

Zika virus (ZIKV) infection during pregnancy can cause significant problems, particularly congenital Zika syndrome. Nevertheless, the potential deleterious consequences and associated mechanisms of transfusion-transmitted ZIKV infection on pregnant individuals and their fetuses and babies have not been investigated. Here we examined transmissibility of ZIKV through blood transfusion in ZIKV-susceptible pregnant A129 mice. Our data showed that transfused-transmitted ZIKV at the early infection stage led to significant viremia and broad tissue tropism in the pregnant recipient mice, which were not seen in those transfused with ZIKV-positive (ZIKV^+^) plasma at later infection stages. Importantly, pregnant mice transfused with early-stage, but not later stages, ZIKV^+^ plasma also exhibited severe placental infection with vascular damage and apoptosis, fetal infection and fetal damage, accompanied by fetal and pup death. Overall, this study suggests that transfusion-related transmission of ZIKV during initial stage of infection, which harbors high plasma viral titers, can cause serious adverse complications in the pregnant recipients and their fetuses and babies.

## Introduction

Zika virus (ZIKV), a flavivirus in the *Flaviviridae* family, was first identified in rhesus monkeys in 1947 (Dick et al., 1952; Wikan and Smith, 2016). Similar to other flaviviruses, including dengue virus, yellow fever virus, West Nile virus, and Japanese encephalitis virus, ZIKV is a positive-stranded RNA virus, whose genome encodes structural proteins, including capsid, pre-membrane (prM)/membrane (M), and envelope (E), as well as seven non-structural proteins (Dai et al., 2016; Sirohi et al., 2016; Zhao et al., 2016). ZIKV E protein is a major protein in receptor binding and fusion, and it consists of ectodomain, e.g., domain I (EDI), II (EDII), and III (EDIII), stem region, and transmembrane region (Dai et al., 2016; Kostyuchenko et al., 2016).

Most people infected with ZIKV have no clinical symptoms or only exhibit mild symptoms, without requiring hospitalization. However, ZIKV infection during pregnancy can cause significant problems, particularly congenital Zika syndrome, which involves congenital brain abnormalities, microcephaly at birth, and motor anomalies and epilepsy in infants, as well as other malformations (Melo et al., 2016; Mlakar et al., 2016; Cui et al., 2017; Krauer et al., 2017; Zorrilla et al., 2017; Chimelli et al., 2018; Pessoa et al., 2018). In addition, ZIKV infection is linked to Guillain-Barre syndrome (GBS), a severe neurological disease (Cao-Lormeau et al., 2016; Roze et al., 2017; Salinas et al., 2017; Simon et al., 2018). The association of ZIKV infection with these unexpected diseases has thus brought worldwide attention to study this virus and its pathogenic mechanisms.

Several mouse models are developed to study ZIKV infection and associated pathology, and to evaluate the efficacy of ZIKV vaccines and therapeutics. We and others have shown the susceptibility of type I interferon receptor (IFNAR)-deficient A129 mice, as well as type I and II interferon receptor (IFNAR/IFNGR)-deficient AG129 and AG6 mice, to ZIKV, and identified candidate vaccines and therapeutic agents that protect against ZIKV infection in these mouse models (Aliota et al., 2016; Cui et al., 2017; Du et al., 2017; Wu et al., 2017; Sumathy et al., 2017; Yu et al., 2017; Espinosa et al., 2018; Tai et al., 2018; Jiang and Du, 2019).

As seen with other mosquito-borne flaviviruses (Harrington et al., 2003; Iwamoto et al., 2003; Wilder-Smith et al., 2009; Aubry et al., 2015), ZIKV has acquired the ability to infect humans via blood or blood components (Barjas-Castro et al., 2016; Motta et al., 2016; Musso et al., 2016; Benjamin, 2017; Bloch et al., 2018). ZIKV RNA was identified in blood donations, and the risk of ZIKV transmission by blood donations does exist, even in the regions with low disease circulation (Galel et al., 2017; Williamson et al., 2017; Magnus et al., 2018). Nevertheless, the transmissibility of ZIKV through blood transfusion and associated pathogenic mechanisms, particularly in pregnant women and their fetuses or babies, are poorly understood. The present study aimed to answer these questions in ZIKV-susceptible pregnant A129 mice. Our data have demonstrated that transfusion with plasma from high-dose, early-stage ZIKV infection led to significant viremia and broad tissue tropism, particularly brain, in pregnant mice. In addition, such transfusion-transmitted ZIKV infection also resulted in severe placental vascular damage, apoptosis, and inflammation, as well as fetal damage and fetal and pup demise. In contrast, transfusion with ZIKV-positive (ZIKV^+^) plasma at later stages of ZIKV infection, which contained low-titer or no ZIKV, did not enable significant ZIKV replication and/or congenital Zika infection.

## Materials and Methods

### Ethics Statement

Adult (6–8-week-old) and pregnant [8–12-week-old, embryonic day (E7–9 and E10–12)] A129 mice were used in the study. The animal studies were carried out in strict accordance with the recommendations in the Guide for the Care and Use of Laboratory Animals of the National Institutes of Health. The protocols were approved by the Committee on the Ethics of Animal Experiments of the New York Blood Center (Permit Numbers: 344.00 and 345.01).

### Virus and Plasma at Different Stages After ZIKV Infection

**FIGURE 1:**
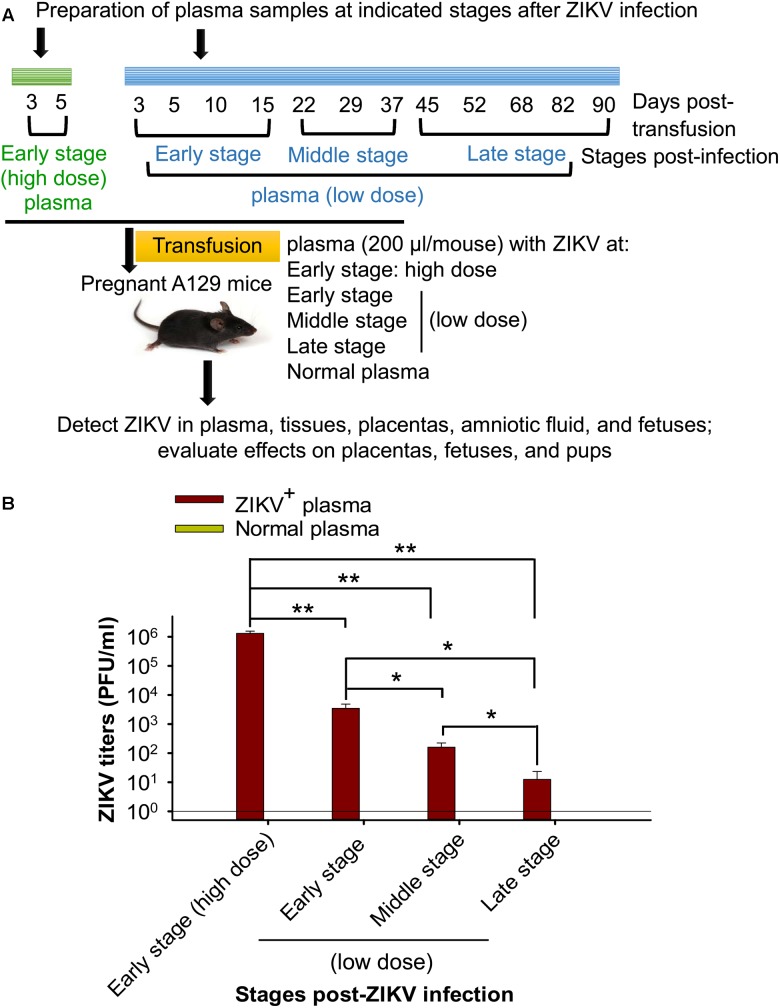
Sample preparation and experimental design. **(A)** Schematic diagram of plasma preparation and experimental procedure. Adult A129 mice were transfused with naive mouse plasma inoculated with or without a non-lethal, low-dose of ZIKV (∼90 PFU). Blood was then collected at the indicated time points to prepare for the pooled plasma at early, middle, and late stages post-ZIKV infection (low dose, shown in blue). In addition, adult A129 mice were transfused with naive mouse plasma with or without ZIKV (∼10^4^ PFU), and blood was collected on days 3 and 5 post-transfusion for preparation of pooled plasma containing high-titer ZIKV at early infection stage (early stage: high dose, shown in green). The above pooled plasma at different stages post-ZIKV infection was transfused to pregnant A129 mice (200 μl/mouse, 6 mice/group) for subsequent experiments. **(B)** Detection of ZIKV titers of above pooled plasma from different infection stages by plaque assay. The ZIKV titers are expressed as PFU/ml, based on which each mouse receiving plasma containing specific ZIKV titer at early stage (high dose), early-, middle-, and late-stages (low dose) was calculated. ^∗^ and ^∗∗^ indicate significant differences between early stage (high dose) and other stages, between low dose of early stage and middle or late stage, or between low dose of middle and late stages, of ZIKV infection. The data are presented as means ± s.e.m of duplicate wells. The experiments were repeated twice, and similar results were obtained. Normal plasma: control plasma from A129 mice transfused with normal plasma without ZIKV (0 PFU).

To understand transmissibility of ZIKV through blood transfusion and determine which stage(s) of ZIKV infection after ZIKV^+^ plasma transfusion that will not cause severe consequences to the pregnant recipients, plasma from early, middle, and late stages of ZIKV infection was prepared (Figure 1). A contemporary ZIKV strain, R103451, which has demonstrated effective infectivity in A129 mice (Tai et al., 2018), was used in the study. To set up ZIKV^+^ plasma transfusion and eliminate potential effects of other factors, such as preexisting antibodies, in the plasma to be transfused, plasma from naive A129 mice was manually added with a non-lethal, low-dose ZIKV (∼90 plaque forming unit: PFU), and transfused into adult A129 mice (200 μl/mouse, 18 mice/group) via retro-orbital injection. Here a non-lethal ZIKV dose was used to maintain mouse survival during the long-term observation period. Plasma was collected from the mice at defined time points after transfusion, and pooled at early, middle, and late stages of infection for subsequent transfusion experiments. To prepare plasma at early-stage ZIKV infection containing a high ZIKV titer (early stage: high dose), adult A129 mice was transfused with plasma (200 μl/mouse, 18 mice/group) with manually added ZIKV (∼10^4^ PFU), and plasma was collected on days 3 and 5 after transfusion. Plasma from A129 mice transfused with normal plasma (200 μl/mouse, 12 mice/group) was collected for control experiments.

### Plasma Transfusion, Sample Collection and Evaluation

Three experiments were designed for evaluating potential consequences of transfusion with plasma containing ZIKV at different infection stages to pregnant mice. First, the aforementioned plasma collected from mice at early, middle, and late stages of ZIKV infection with calculated ZIKV titers was transfused into pregnant (E10–12) A129 mice (200 μl/mouse, 6 mice/group) via retro-orbital injection. Six days later, plasma, tissues, placentas, fetuses, and amniotic fluid were collected for detecting viral replication and for subsequent experiments described below. Second, pregnant (E7–9 and E10–12) A129 mice were transfused with early-stage (high dose) ZIKV^+^ plasma (200 μl/mouse, 6 mice/group), and uteri and placentas were collected 6 days post-transfusion to compare for placental morphology and fetal changes. Third, pregnant (E10–12) A129 mice transfused with early-stage (high dose) ZIKV^+^ plasma (200 μl/mouse, 6 mice/group) were counted for newborn pups with or without survival. A129 mice transfused with normal plasma (200 μl/mouse, 6 mice/group) were used as controls.

### Quantitative Reverse Transcriptase PCR (qRT-PCR)

ZIKV RNA copies in the collected samples were detected by qRT-PCR (Tai et al., 2018). Briefly, RNA was extracted using QIAamp MinElute Virus Spin Kit (for plasma and amniotic fluid) (Qiagen) and RNeasy Mini Kit (for tissues) (Qiagen), and quantified using one-step qRT-PCR in the presence of Power SYBR Green PCR Master Mix, MultiScribe Reverse Transcriptase, and Ambion^TM^ RNase Inhibitor (Thermo Fisher Scientific) in ViiA 7 Master Cycler PCR System (Thermo Fisher Scientific). The forward (5′-TTGGTCATGATACTGCTGATTGC-3′) and reverse (5′-CCTTCCACAAAGTCCCTATTGC-3′) primers were used for the amplification. To establish a standard curve of qRT-PCR, the M-E genes of ZIKV (strain R103451, 141–1800 bp) were amplified and cloned into pCR2.1-TOPO vector (Thermo Fisher Scientific) to construct a recombinant plasmid. This plasmid was serially diluted at 10-fold, and a linear standard curve at 10^2^ to 10^10^ RNA copies (correlation coefficient *R^2^* value > 0.99; detection limit: ∼10^2^ RNA copies) was selected to calculate ZIKV RNA in the plasma-transfused mouse samples. ZIKV RNA was purified from about 40 μl (for plasma or amniotic fluid) or 40 mg (for tissues) of transfused samples, so the detection limit per milliliter (ml) or gram (g) was about 2.5 × 10^3^ RNA copies.

### Measurement of ZIKV Titers

Plaque-forming assay was carried out to measure ZIKV titers in collected samples (Wu et al., 2017; Tai et al., 2018). Briefly, diluted plasma, amniotic fluid, and tissue lysates were transferred onto Vero E6 cells (ATCC) and incubated at 37°C for 1 h. The cells were further overlaid with DMEM (Thermo Fisher Scientific) containing 1% carboxymethyl cellulose (MilliporeSigma) and 2% FBS (Atlanta Biologicals), cultured at 37°C for four to five days, and stained with 0.5% crystal violet (MilliporeSigma). Viral titers are presented as PFU/ml or PFU/g of test samples.

### Immunofluorescence Staining

Harvested maternal brain and placentas were fixed in 4% paraformaldehyde (MilliporeSigma), embedded in paraffin, and sectioned (Sapparapu et al., 2016; Yuan et al., 2017). The tissue slides were deparaffinized, blocked with 2% BSA (MilliporeSigma) for 30 min, and then incubated with human anti-ZIKV (EDIII-specific monoclonal antibody (mAb) ZV-64, 1:200, Absolute Antibody) and rabbit anti-vimentin antibody (1:300, Abcam), respectively, at 4°C overnight. For caspase-3 staining, the deparaffinized tissue slides were fixed and permed with FIX and PERM Cell Permeabilization Kit (Thermo Fisher Scientific), then blocked and incubated with rabbit anti-active caspase-3 antibody (1:200, Abcam) as described above. After washing with PBS, the slides were incubated with anti-human FITC (for ZIKV) or anti-rabbit Alexa Fluor^®^ 647 (1:300, Abcam; for vimentin and caspase-3)-conjugated antibodies for 1 h. The slides were counter-stained with DAPI (4′,6-diamidino-2-phenylindole, 300 nM, Thermo Fisher Scientific) for nuclei for 5 min, and mounted in a VectaMount Permanent Mounting Medium (Vector Laboratories). All slides were imaged on confocal microscope (Zeiss LSM 880). Images were prepared using Adobe Photoshop and ZEN software, and the fluorescent signals were quantified by ImageJ software for the relative intensity (particle analysis) using a standard protocol (Wu et al., 2003). Specifically, the pictures were set up image type at 8-bit, and those from the experiment and control groups were adjusted to the same thresholds. The fluorescence intensity of the particles was then analyzed to get positive counts of each picture.

### Transmission Electron Microscope (TEM)

Tissues were dissected in PBS, and 3 mm^3^ fragments were fixed with 2.5% glutaraldehyde (MilliporeSigma) and 2% paraformaldehyde in sodium cacodylate buffer (0.1 M, MilliporeSigma) for at least 2 h. After fixation, samples were washed three times in sodium cacodylate buffer (0.1 M) and post-fixed by 2% OsO4 (MilliporeSigma) for 1 h. Samples were then washed and dehydrated using a series of ethanol concentrations (50–100%) with a final wash of propylene oxide (MilliporeSigma). Samples were embedded in plastic (Epon 812, EMS) and prepared for sectioning. Ultrathin sections were contrasted with UranyLess (EMS) and lead citrate (MilliporeSigma), and analyzed under the Tecnai G2 Spirit TEM.

### Detection of Inflammatory Cytokines and Chemokines

Inflammatory cytokines and chemokines were measured in collected placentas and amniotic fluid using Mouse Inflammatory Cytokines Multi-Analyte ELISArray Kit and Mouse Common Chemokines Multi-Analyte ELISArray Kit according to the manufacturer’s instructions (Qiagen). Briefly, mouse placental lysates and amniotic fluid were incubated with respective cytokine and chemokine capture antibodies pre-coated in the ELISA plates for 2 h at room temperature. After three washes, the plates were sequentially incubated with detection antibody for 1 h, and Avidin-HRP conjugate for 30 min at room temperature. The plates were then, respectively, incubated with development solution and stop solution to stop the reaction. Absorbance at 450 nm was measured by ELISA plate reader (Tecan).

### Statistical Analysis

The values are presented as means with standard error (s.e.m). Statistical significance among different groups was calculated using GraphPad Prism Statistical Software. For data comparing viral RNA copies, viral titers, and fluorescence staining (shown on a log-based scale), Welch’s *t*-test was used, and for other data, two-tailed Student’s *t*-test was applied. ^∗^, ^∗∗^ and ^∗∗∗^ represent *P* < 0.05, *P* < 0.01, and *P* < 0.001, respectively.

## Results

### Preparation of Plasma Samples at Different Stages of ZIKV Infection

To prepare plasma at early, middle, and late infection stages with low-dose ZIKV, plasma containing ZIKV at a non-lethal dose (∼90 PFU), which was previously identified via intraperitoneal (i.p.) route (Tai et al., 2018), was transfused into A129 mice, and blood was collected and pooled for plasma from defined time points after transfusion (Figure 1A) to detect for ZIKV viral titers. To prepare plasma at early stage (high dose) of ZIKV infection, mice were inoculated with a high, lethal-dose of ZIKV [∼10^4^ PFU: previously identified through i.p. route (Tai et al., 2018)] and plasma collected at early stage post-ZIKV infection (day 3 and 5 after transfusion) (Figure 1A). This plasma contains significantly higher ZIKV titers than the plasma collected from mice receiving non-lethal, low-dose ZIKV at early, middle, and late stages of infection. In addition, ZIKV titers in the plasma of mice from early and middle infection stages (low dose) were also significantly higher than those from later infection stages (low dose) (Figure 1B).

### Transfusion of High-Dose Early-Stage ZIKV^+^ Plasma to Pregnant A129 Mice Caused Significant Viremia and Broad Tissue Tropism, Including Brain

To investigate tissue distribution of ZIKV in pregnant A129 mice following transfusion with ZIKV^+^ plasma, we performed the following analyses.

**FIGURE 2:**
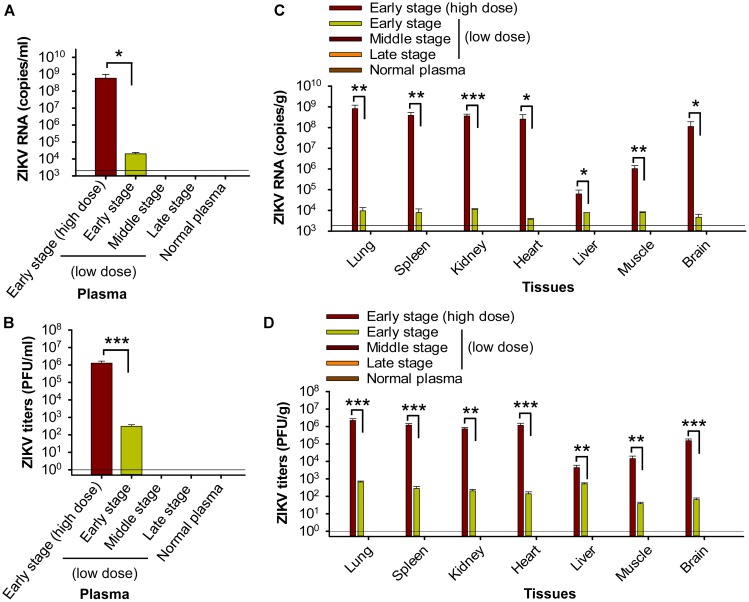
Transfusion of pregnant A129 mice with early-stage (high dose) ZIKV^+^ plasma resulted in significant viremia and broad tissue tropism. Pregnant (E10–12) A129 mice were transfused with ZIKV^+^ plasma from early, middle, and late stages, respectively, and plasma and tissues were collected 6 days post-transfusion for below tests. Plasma ZIKV RNA **(A)** and ZIKV titers **(B)** were detected by qRT-PCR and plaque assay, respectively. Tissue ZIKV RNA **(C)** and viral titers **(D)** were detected by qRT-PCR and plaque assay, respectively. ^∗^, ^∗∗^, and ^∗∗∗^ indicate significant differences between early stage (high dose) and early stage (low dose) post-transfusion with ZIKV^+^ plasma. The data are presented as means ± s.e.m (*n* = 6 mice/group). In **(A)** and **(C)**, the limit of detection, shown as the horizontal lines, was about 2.5 × 10^3^ copies/ml (for **A**) or 2.5 × 10^3^ copies/g (for **C**) of ZIKV RNA. This was determined based on a detection limit of 10^2^ copies in a linear standard curve (at serial dilutions of 10^2^ to 10^10^ copies) of ZIKV RNA, as described in Materials and Methods. In **(B)** and **(D)**, the detection limit was about 1 PFU/ml (for **B**) or 1 PFU/g (for **D**). The experiments were repeated twice, and similar results were obtained. Normal plasma: control plasma from A129 mice transfused with normal plasma without ZIKV.

First, we measured viral load on day 6 post-transfusion, an optimal time point for detecting viral RNA levels and viral titers in tissues and blood (Dowall et al., 2016). We found that pregnant mice (E10–12) receiving plasma at the early-stage ZIKV infection containing a high titer (∼2.6 × 10^5^ ± 5 × 10^4^ PFU) of ZIKV (early stage: high dose) exhibited severe ZIKV viremia, with significantly higher levels of ZIKV RNA (Figure 2A) and ZIKV titers (Figure 2B) than those receiving plasma containing a lower titer, early-stage (low dose) ZIKV^+^ (∼690 ± 285 PFU). In contrast, pregnant mice receiving middle- (∼32 ± 13 PFU) and late-stage (∼3 ± 2 PFU) (low dose) ZIKV^+^ plasma had undetectable levels of viremia similar to normal plasma-transfused mice (Figures 2A,B). These data reveal that establishment of transfusion-transmitted ZIKV infection in pregnant mice might occur only with early-stage infected plasma containing high-dose ZIKV.

**FIGURE 3:**
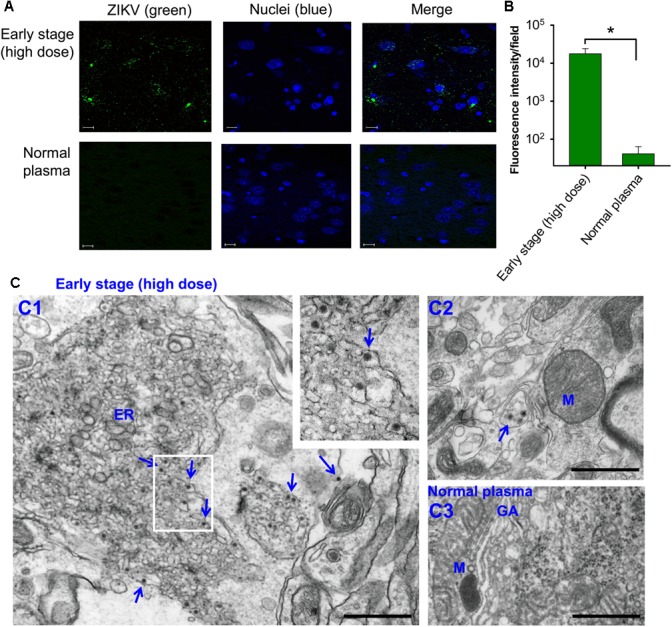
Identification of ZIKV particles in brain tissues of pregnant A129 (E10–12) mice transfused with early-stage (high dose) ZIKV^+^ plasma. **(A)** Immunofluorescence staining of brain tissues collected 6 days post-transfusion. Brain sections were stained for ZIKV (green) using ZIKV EDIII-specific mAb ZV-64, and representative images are listed. Nuclei were stained with DAPI, and are shown in blue. Magnification: 63X; Scale bar: 10 μm. **(B)** Quantification of ZIKV staining in **(A)** was calculated by ImageJ software for the relative intensity (particle analysis) of ZIKV staining with fluorescent signals. The data are presented as mean fluorescence intensity (e.g., ZIKV^+^ staining) in each field ± s.e.m (*n* = 6, “*n*” indicates numbers of image from different brain tissues), and ^∗^ indicates significant differences between early stage (high dose) and normal plasma groups. **(C)** TEM analysis of above brain tissues. Microphotographs of ZIKV particles formed in brain tissues of mice transfused with early-stage (high dose) ZIKV^+^ plasma (C1–C2) or normal plasma (C3). Short arrows indicate ZIKV particles. ER, endoplasmic reticulum; M, mitochondria; GA, golgi apparatus. Scale bar: 500 nm. Normal plasma groups (A–C) indicate control mice transfused with normal plasma without ZIKV.

Second, analysis of tissues, including brain, on day 6 post-transfusion demonstrated that pregnant (E10–12) mice transfused with early-stage high-dose ZIKV^+^ plasma had significantly higher ZIKV RNA (Figure 2C) and ZIKV titers (Figure 2D) in lung, spleen, kidney, heart, liver, muscle, and brain than mice receiving plasma from early stage (low dose) of ZIKV infection. In contrast, ZIKV RNA and ZIKV titers were undetectable in the above tissues of mice transfused with middle- and late-stage (low dose) ZIKV^+^ plasma, or normal plasma (Figures 2C,D). The presence of ZIKV in brain tissues of pregnant mice transfused with early-stage high-dose ZIKV^+^ plasma was confirmed by immunofluorescence staining of ZIKV E protein using a ZIKV EDIII-specific mAb (Figure 3A), which showed significantly more ZIKV staining than in those of mice transfused with normal plasma (Figure 3B), as well as by TEM analysis, which revealed multiple particles (∼50–80 nm) in highly vacuolated cells, indicative of ZIKV infection (Figure 3C).

The above data indicate that transfusion of ZIKV^+^ plasma from high-dose early-stage, but not later stages, of ZIKV infection leads to severe complications in pregnant A129 recipient mice, causing high viremia and broad tissue tropism, particularly brain.

### Transfusion of High-Dose Early-Stage ZIKV^+^ Plasma to Pregnant A129 Mice Resulted in Severe Placental Infection, as Well as Vascular Damage and Apoptosis in Placentas

To further investigate whether transfusion-transmitted ZIKV causes placental infection and damage, pregnant (E10–12) A129 mice were transfused with ZIKV^+^ plasma from different infection stages, and placentas were analyzed on day 6 post-transfusion.

**FIGURE 4:**
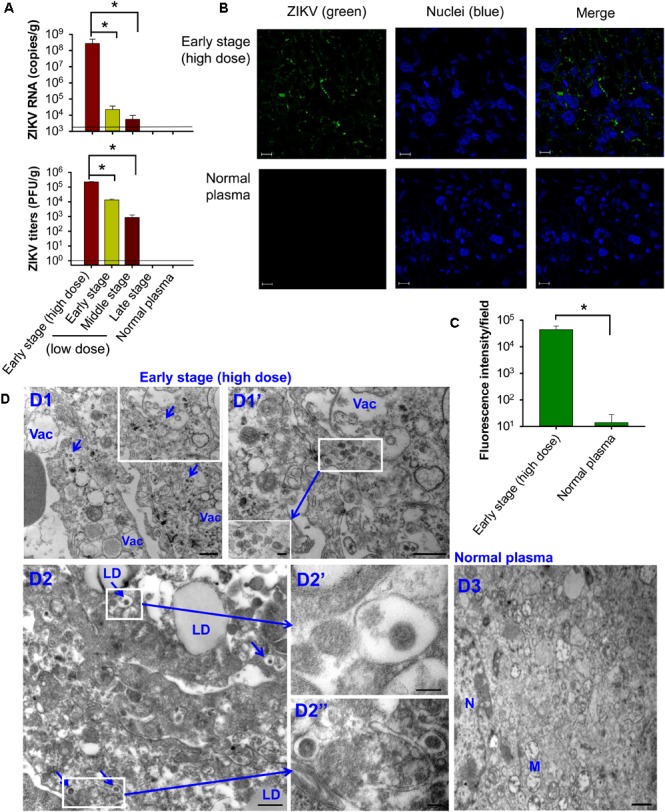
Transfusion of pregnant A129 mice with early-stage (high dose) ZIKV^+^ plasma led to significant placental infection. Pregnant (E10–12) A129 mice were transfused with ZIKV^+^ plasma from early, middle, and late stages, respectively, and placentas were collected 6 days after transfusion for below tests. Detection of ZIKV RNA by qRT-PCR (**A**, top) and ZIKV titers by plaque assay (**A**, bottom). The data are presented as means ± s.e.m (*n* = 6 mice/group). ^∗^ indicates significant differences between early stage (high dose) and early or middle stages (low dose). The detection limit, shown as the horizontal lines, for qRT-PCR and plaque assay was about 2.5 × 10^3^ RNA copies/g and 1 PFU/g, respectively. **(B)** Immunofluorescence staining of placental tissues of pregnant mice transfused with early-stage (high dose) ZIKV^+^ plasma. ZIKV (green) was stained using ZIKV EDIII-specific mAb ZV-64. Nuclei were stained with DAPI, and are shown in blue. Representative images are listed. Magnification: 63X; Scale bar: 10 μm. **(C)** Quantification of ZIKV staining in (B) was calculated by ImageJ software for the relative intensity (particle analysis) of ZIKV staining with fluorescent signals. The data are presented as mean fluorescence intensity (e.g., ZIKV^+^ staining) in each field ± s.e.m (*n* = 6, “*n*” indicates numbers of image from different placentas). ^∗^ indicates significant differences between early stage (high dose) and normal plasma groups. **(D)** TEM analysis of placental tissues collected from the mice transfused with early-stage (high dose) ZIKV^+^ plasma. D1–D2, microphotographs show ZIKV particles in the placentas of ZIKV^+^ plasma-transfused mice. Infected cells were highly vacuolated and abundant with lipid droplets. Short arrows indicate ZIKV particles. D3, placental tissues collected from normal plasma-transfused mice. Vac, vacuole; LD, lipid droplet; M, mitochondria; N, nuclei. Scale bar: 500 nm for D1, D1”, D2, and D3, and 100 nm for D1’ insertion, D2’, and D2”. Normal plasma: control plasma from A129 mice transfused with normal plasma without ZIKV.

Significantly higher levels of ZIKV RNA (Figure 4A, top) and viral titers (Figure 4A, bottom) were detected in the placentas of mice transfused with early-stage (high dose) ZIKV^+^ plasma than those receiving ZIKV^+^ plasma at early and middle stages (low dose) of ZIKV infection, whereas both ZIKV RNA and viral titers were undetectable in the placentas of mice transfused with late-stage (low dose) ZIKV^+^ plasma or normal plasma. Immunofluorescence staining also demonstrated strong signals with ZIKV-specific mAb in the placentas of early-stage (high dose) ZIKV^+^ plasma-transfused mice (Figure 4B), and greater numbers of ZIKV^+^ signals than those of control mice (Figure 4C). TEM analysis of infected placental tissues revealed high numbers of particles (∼50–80 nm) in the early-stage (high dose) ZIKV^+^ plasma-transfused placental tissues. In addition, the cytoplasm of placental cells of mice transfused with ZIKV^+^ plasma, but not normal plasma control, had an abundance of vacuoles and large lipid droplets (Figure 4D).

**FIGURE 5:**
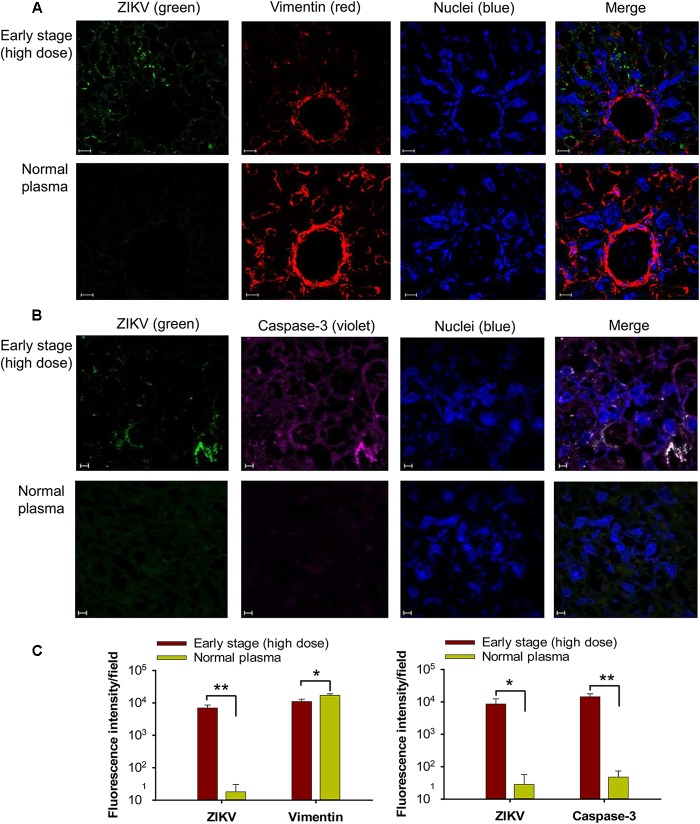
Transfusion of pregnant A129 mice with early-stage (high dose) ZIKV^+^ plasma caused vascular damage and apoptosis in placentas. Placentas were collected from pregnant (E10–12) A129 mice transfused with early-stage (high dose) ZIKV^+^ plasma 6 days after transfusion, and used for below tests. Representative images of immunofluorescence staining of vimentin **(A)** and activated form of caspase-3 **(B)** in placentas are shown. ZIKV (green), vimentin (red), and activated caspase-3 (violet) were stained with anti-ZIKV, anti-vimentin, and anti-active caspase-3 antibodies, respectively. Nuclei were stained with DAPI, and are shown in blue. Magnification: 63X. Scale bar: 10 μm. **(C)** Quantification of vimentin and caspase-3 staining in **(A)** and **(B)** by ImageJ software as described above. The data are presented as mean fluorescence intensity for ZIKV^+^, vimentin^+^, or caspase-3^+^ staining in each field ± s.e.m (*n* = 6, “*n*” indicates numbers of image from different placentas). ^∗^ and ^∗∗^ indicate significant differences between early stage (high dose) and normal plasma groups. Normal plasma: control plasma from A129 mice transfused with normal plasma without ZIKV.

To identify potential mechanisms of placental damage, placentas from pregnant mice transfused with high-dose early-stage ZIKV^+^ plasma were also investigated for vascular damage and apoptosis. By immunofluorescence microscopy, diminished staining for vimentin, a marker for fetal capillary endothelium and fetal blood vessels in mouse placentas (Sapparapu et al., 2016; Miner et al., 2016), was detected in placentas from mice transfused with ZIKV^+^ plasma, with significantly reduced numbers of vimentin^+^ signals than those of the mice transfused with normal plasma (Figures 5A,C), suggesting damaged vasculature associated with early-stage ZIKV^+^ plasma transfusion. The placentas of mice transfused with high-dose early-stage ZIKV^+^ plasma also stained positive for activated form of caspase-3, an apoptotic marker (Yuan et al., 2017), with significantly higher numbers than those from normal plasma-transfused mice (Figures 5B,C), demonstrating considerable cell death, consistent with reduced vimentin expression.

Altogether, these data indicate severe placental infection, vascular damage, and apoptosis following transfusion of pregnant A129 mice with early-stage ZIKV^+^ plasma containing high titer (∼2.6 × 10^5^ ± 5 × 10^4^ PFU) of infectious particles.

### Transfusion of High-Dose Early-Stage ZIKV^+^ Plasma to Pregnant A129 Mice Resulted in Severe Fetal Infection and Damage, Leading to Fetal and Pup Death

**FIGURE 6:**
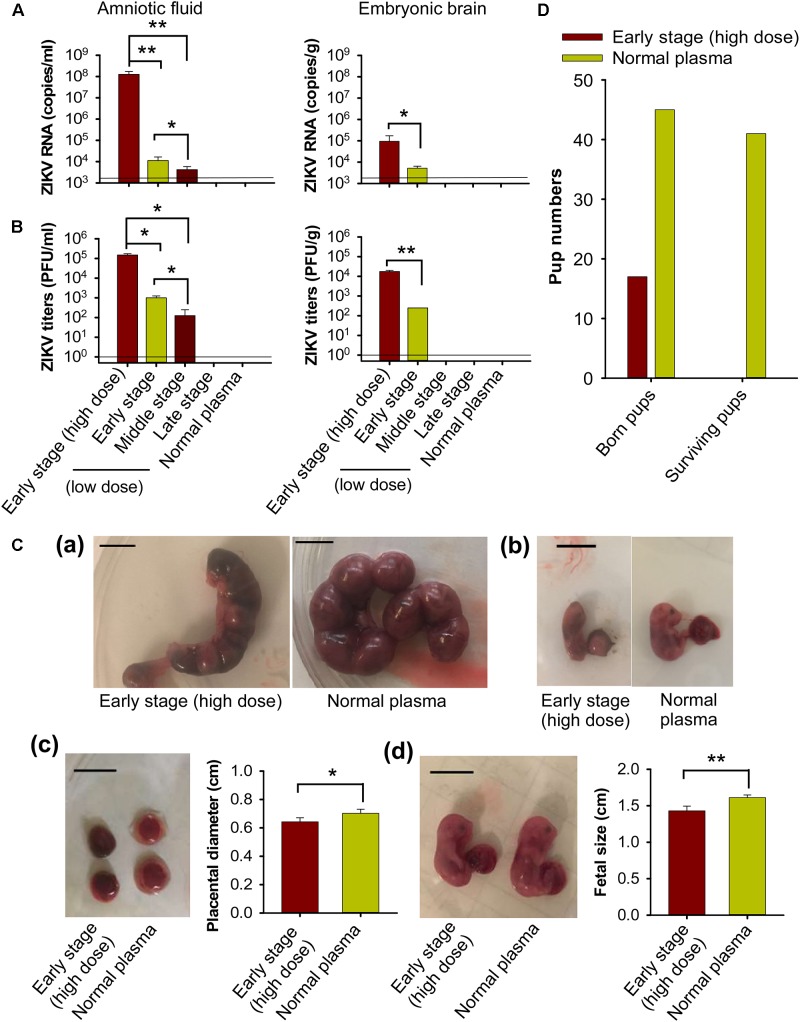
Transfusion of pregnant A129 mice with early-stage (high dose) ZIKV^+^ plasma led to significant fetal infection and fetal and pup death. Pregnant (E10–12) A129 mice were transfused with ZIKV^+^ plasma from early, middle, and late stages, respectively. ZIKV RNA and ZIKV titers were detected by qRT-PCR **(A)** and plaque assay **(B)** in amniotic fluid and embryonic brain collected 6 days post-transfusion. The detection limit, shown as the horizontal lines, was about 2.5 × 10^3^ RNA copies/ml and 1 PFU/ml (for amniotic fluid), or 2.5 × 10^3^ RNA copies/g and 1 PFU/g (for embryonic brain), respectively, for qRT-PCR and plaque assay. ^∗^ and ^∗∗^ indicate significant differences between early stage (high dose) and early or middle stages (low dose), or between early and middle stages (low dose). The data are presented as means ± s.e.m (*n* = 6 mice/group). **(C)** Morphology of mouse uteri and placentas and conditions of fetuses 6 days after transfusion of early-stage (high dose) ZIKV^+^ plasma. **(a)** E13–15 uteri from pregnant mice transfused at E7–9. **(b)** Representative images of fetuses in **(a)**. **(c)** E16–18 placentas from pregnant mice transfused at E10–12. **(d)** Representative images of E16–18 fetuses in **(c)**. Scale bar: 1 cm. Placental diameter in **(c)** and fetal size in **(d)** are shown. ^∗^ and ^∗∗^ indicate significant differences between early stage (high dose) and normal plasma groups. The data are presented as means ± s.e.m (*n* = 6 mice/group). **(D)** Total born and surviving pups 24 h after birth from pregnant (E10–12) mice transfused with early-stage (high dose) ZIKV^+^ plasma. Normal plasma: control plasma from A129 mice transfused with normal plasma without ZIKV.

To test the impact of transfusion-transmitted ZIKV infection during pregnancy on fetuses and newborn pups, we first measured ZIKV in fetal amniotic fluid and embryonic brain tissue from pregnant (E10–12) A129 mice 6 days after transfusion with ZIKV^+^ plasma. We found significantly higher ZIKV RNA (Figure 6A) and viral titers (Figure 6B), in amniotic fluid and embryonic brain of the pregnant mice transfused with early-stage (high dose) ZIKV^+^ plasma than with plasma of early and/or middle stages (low dose) of ZIKV infection. There were also significantly higher ZIKV RNA and viral titers in amniotic fluid of the mice transfused with early-stage (low dose) than middle-stage (low dose) ZIKV^+^ plasma. In contrast, ZIKV RNA and viral titers were undetectable in aforementioned samples of the mice transfused with other stages (low dose) of ZIKV^+^ plasma, or normal plasma (Figures 6A,B).

To investigate effects of transfusion-transmitted ZIKV infection during gestation, pregnant A129 mice at E7–9 and E10–12 were transfused with early-stage (high dose) ZIKV^+^ plasma, and then evaluated for uterus and placental morphology and fetus conditions 6 days after transfusion. All fetuses from ZIKV^+^ plasma-transfused pregnant (E7–9) mice died in uteri and had experienced severe resorption (Figure 6Ca). In addition, these fetuses displayed significant growth restriction compared to those from the pregnant mice transfused with normal plasma (Figure 6Cb). Also, placentas from pregnant (E10–12) mice transfused with ZIKV^+^ plasma presented obvious congestion, with significantly smaller diameter than those of the mice transfused with normal plasma (Figure 6Cc). Particularly, the size of the fetuses from ZIKV^+^ plasma-transfused pregnant (E10–12) mice was significantly smaller than that of the fetuses from normal plasma-transfused mice (Figure 6Cd), suggesting significant fetal growth restriction during pregnancy in the ZIKV^+^ plasma-transfused mice.

We further monitored the survival of newborn pups from pregnant (E10–12) A129 mice transfused with early-stage (high dose) ZIKV^+^ plasma. Fewer pups were born to ZIKV^+^ plasma-transfused pregnant mice than to normal plasma-transfused mice, and all pups from the former group died by 24 h after birth (Figure 6D).

The above data demonstrate that while transfusion of pregnant A129 mice with low-dose ZIKV^+^ plasma at early (∼690 ± 285 PFU), middle (∼32 ± 13 PFU), and late (∼3 ± 2 PFU) stages of infection led to low to no ZIKV replication in fetuses, transfusion with early-stage (high dose) ZIKV^+^ (∼2.6 × 10^5^ ± 5 × 10^4^ PFU) plasma caused severe congenital fetal infection, fetal damage, and reduced fetal growth, leading to fetal and pup death.

### Transfusion of High-Dose Early-Stage ZIKV^+^ Plasma to Pregnant A129 Mice Resulted in Inflammation With Increased Cytokines and Chemokines

**FIGURE 7:**
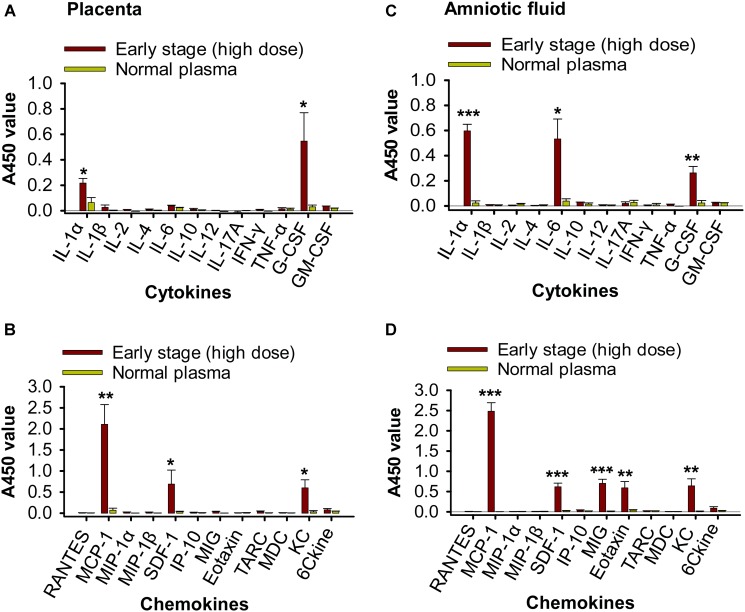
Transfusion of pregnant A129 mice with early-stage (high dose) ZIKV^+^ plasma led to significant inflammation. Placentas **(A,B)** and amniotic fluid **(C,D)** collected from pregnant (E10–12) A129 mice 6 days post-transfusion of early-stage (high dose) ZIKV^+^ plasma were detected by Multi-Analyte ELISArray Kits for inflammatory cytokines **(A,C)** and chemokines **(B,D)**. ^∗^, ^∗∗^, and ^∗∗∗^ indicate significant differences between early stage (high dose) and normal plasma groups, and the data are presented as means ± s.e.m (*n* = 6 mice/group). Normal plasma: control plasma from A129 mice transfused with normal plasma without ZIKV.

To evaluate whether transfusion-transmitted ZIKV causes inflammation to pregnant mice and their fetuses, placentas and fetal amniotic fluid from pregnant (E10–12) A129 mice 6 days after transfusion were tested for inflammatory cytokines and chemokines using Multi-Analyte ELISArray Kits. Significantly higher expression of IL-1α, G-CSF, MCP-1 (CCL-2), SDF-1 (CXCL-12), and KC (CXCL-1) was identified in the ZIKV^+^ plasma (early-stage, high dose)-transfused mouse placentas than those of the mice transfused with normal plasma (Figures 7A,B). In addition to the cytokines and chemokines described above, fetal amniotic fluid of the mice transfused with ZIKV^+^ plasma also demonstrated significantly increased production of inflammatory cytokine IL-6 and chemokines MIG (CXCL-9) and Eotaxin (CCL-11), as compared with that of the normal plasma-transfused mice (Figures 7C,D). These data suggest that early-stage (high dose) ZIKV^+^ plasma transfusion may lead to significant inflammation in the pregnant mice, and that fetuses might experience a more severe inflammatory response than maternal placentas.

## Discussion

ZIKV may be transmitted through blood transfusion. ZIKV RNA was detected in asymptomatic blood donors during the ZIKV outbreak, posing a serious threat to blood safety (Musso et al., 2016; Bierlaire et al., 2017; Slavov et al., 2017; Willyard, 2017). Here, we used ZIKV-susceptible pregnant A129 mice to study transmissibility of ZIKV through transfusion and evaluate viremia, tissue tropism, placental infection and damage, and associated pathogenic mechanisms, as well as fetal damage and fetal and pup death. Overall, the data suggest that transfusion-transmitted ZIKV from early-stage infection can cause significant viremia, broad tissue tropism, and inflammation, with devastating effects during pregnancy, particularly on the fetuses.

To establish transfusion process with ZIKV^+^ plasma, minimize potential impact of preexisting antibodies or other factors in the transfused plasma, and maintain the recipient mice to survive during the observation period, we manually inoculated normal plasma with a contemporary ZIKV strain (R103451) at non-lethal, low-dose (e.g., ∼90 PFU) (Tai et al., 2018), transfused it to A129 mice, and collected plasma at early, middle, and late ZIKV infection stages after transfusion to test ZIKV titers. Surprisingly, viremia was detectable in the transfused mouse plasma after 45 days, with early-stage infection having the highest ZIKV titer. It has been shown that viral load in human maternal sera can be detectable for 14 weeks, and that ZIKV RNAs are detected in maternal sera 8 weeks after the onset of clinical symptoms, which potentially result from viral replication in the fetuses or placentas where the latter might serve as a reservoir (Driggers et al., 2016; Suy et al., 2016). Here we speculate that persistent ZIKV viremia post non-lethal, low-dose ZIKV^+^ plasma transfusion might be partially due to viral replication in organs that could act as potential reservoirs.

To determine which stages of ZIKV infection with the identified viral titers that will not cause problems to the transfused pregnant recipients, plasma collected above at early, middle, and late stages of ZIKV infection was transfused into pregnant A129 mice. We found that significantly higher viremia was identified in the pregnant mice receiving early-stage (high-dose) ZIKV^+^ plasma than other stages of ZIKV^+^ plasma, suggesting that transfusion-transmitted ZIKV infection at early stage containing high-titer ZIKV will lead to rapid ZIKV replication. Although ZIKV titers were significantly lower in the pregnant mice transfused with plasma at early-stage (low dose) ZIKV infection, it remains possible that such transfusion might have long-term effects.

ZIKV infection may trigger GBS (severe neuropathies) (Savino et al., 2017; Zhou et al., 2017; Hygino da Cruz et al., 2018; Wu et al., 2018) and it is closely associated with congenital Zika diseases (microcephaly and brain abnormalities) in humans (Franca et al., 2016; Shapiro-Mendoza et al., 2017; Zorrilla et al., 2017). ZIKV RNA replication has been identified in human brain and placental tissues (Bhatnagar et al., 2017). Our data show that while pregnant mice had low to no brain and congenital ZIKV infection upon transfusion with early-, middle-, and late-stage plasma containing the low-titer ZIKV, they had significant brain infection with identified ZIKV particles in the infected brain tissue after receiving the high-titer ZIKV^+^ plasma. Notably, such transfusion also caused severe ZIKV replication in the placentas, amniotic fluid, and fetal brain, as well as inflammation in the placentas and fetal amniotic fluid, and had detrimental effects on the placentas, fetuses and newborn babies, causing fetal damage, and fetal and pup death. Overall, since elevated viral titers were associated with increased cell death and damaged vasculature in the placentas, loss of placental integrity in pregnant mice may have been a contributor of increased fetal and newborn mortality.

Clearly, identification of transfusion-transmitted ZIKV infection in brain and placental tissues of pregnant mice, as well as associated fetal damage and pup death by vertical transmission during pregnancy, further reinforce the need for surveillance measures to detect ZIKV in blood products. Our data from mouse studies also suggest that low ZIKV plasma titers might not be associated with establishment of transfusion-transmitted ZIKV infection in brain, placentas, and fetuses. It is thus reassuring that only high-titer ZIKV infection drives complications in pregnant mice and/or their fetuses and pups, suggesting that high risk of transfusion-transmitted ZIKV infection may be restricted to high viral titers at the early stage. Since murine and human placentas present different developmental anatomy and distinct co-expression patterns of certain genes (Georgiades et al., 2002; Soncin et al., 2018), infectious doses required for efficient ZIKV transfusion transmission in mouse and human placentas may differ. Thus, the transfusion studies performed in mice might not be completely consistent in human settings. It is possible that humans receiving ZIKV-containing plasma or blood at different doses, particularly low dose, might have distinct consequences from mice. Nevertheless, considering that murine placentas have paralleling development to the first half of human placental development (through gestational week 16), and that there are still some similarities between human and mouse placentas in terms of structures and cell types (Georgiades et al., 2002; Soncin et al., 2018), the current data provide important information to infection window period and evaluate risk of transfusion-associated transmission by ZIKV through blood products in humans.

Our study has identified potential differences in transfusion-induced ZIKV tissue tropism and/or associated pathology compared to transmission by other routes. For example, ZIKV subcutaneous (S.C.) inoculation leads to detectable virus in peripheral nervous tissues, lymphoid tissues, joints, and uterus at day 7 post-infection (Hirsch et al., 2017). Other studies have shown that ZIKV infection via mosquito bite (e.g., route for human ZIKV infection) alters ZIKV tissue tropism, but the distribution is limited to hemolymphatic tissues, reproductive tract tissues, kidney, and liver (Dudley et al., 2017). More recently, ZIKV infection and congenital Zika syndrome were found to be also associated with critical human congenital heart disease (Angelidou et al., 2018). It is noted that pregnant mice infected with ZIKV through different routes also present variant symptoms, viral replication, and pathology in placentas, fetuses, and pups, and the doses required for efficient viral infection may vary. For instance, pregnant immunocompetent mice, such as BALB/c and C57BL/6, do not generally show significant clinical symptoms, viral replication, and tissue tropism via S.C., I.P., or intravenous (I.V.) routes after high-dose ZIKV infection (≥10^5^ PFU), except for some fetal infection or fetal abnormalities (fetal resorption or growth restriction) (Yu et al., 2017; Caine et al., 2018; Szaba et al., 2018). However, pregnant C57BL/6 or BALB/c mice injected with anti-IFNAR antibodies may have significant viral replication in placentas and fetal heads after I.V. or S.C. infection of ZIKV (10^5^–10^6^ PFU) (Turner et al., 2017; Caine et al., 2018; Li et al., 2018). Particularly, A129 and AG129 mice infected with ZIKV through S.C. or I.P. routes demonstrate severe placental injury, fetal brain injury, shorter skull length, reduced size in fetuses and pups, or fetal and pup demise, with viral doses ranging from 10^2^–10^4^ PFU, focus forming unit (FFU), 50% tissue culture infectious doses (TCID_50_), or 50% cell culture infectious doses (CCID_50_) (Miner et al., 2016; Caine et al., 2018; Julander et al., 2018; Tai et al., 2018). Here we found that transfusion of A129 mice with early-stage ZIKV^+^ (2.6 × 10^5^ PFU) plasma induced broad tissue tropism 6 days post-transfusion, resulting in high viral titers in lung, spleen, kidney, heart, liver, muscle, brain, and placentas, as well as severe fetal infection and damage, accompanying with fetal and pup death. This consequence could be partially contributed to the high-dose ZIKV contained in the transfused plasma. Although other infection stages of plasma with the low-dose ZIKV did not cause significant tissue tropism at short-term (6 days) post-transfusion, ZIKV might replicate slowly but continuously, leading to long-term effects. It should be clarified that the ZIKV tissue tropisms and associated pathology resulting from various infection routes, as described above, did not count for different virus strains used for infection, hosts, age of animals, or time points post-infection. Therefore, comparative studies of viral titers by various routes of ZIKV transmission are needed to determine whether more severe outcome is linked to transfusion-transmitted ZIKV infection, and to develop a more comprehensive profile of ZIKV tropism in all tissues and related outcome to help better understand ZIKV pathogenesis.

To summarize, we report on the transmissibility and pathogenesis of transfusion-transmitted ZIKV infection in ZIKV-susceptible pregnant A129 mice. The data indicate that transfusion of plasma containing ZIKV at early-stage infection harboring high-titer ZIKV has serious consequences for pregnant mice, their fetuses and newborn pups. In contrast, transfusion-associated transmission with plasma from later stages of ZIKV infection, which contained low-titer ZIKV, might not be associated with ZIKV replication and have no serious outcome during pregnancy. Overall, the consequences from transfusion-transmitted ZIKV infection in the pregnant mice are positively correlated with ZIKV titers in the transfused plasma. Taken together, our data may help future design of studies to perform risk assessment of ZIKV transmissibility by blood transfusion in humans.

## Author Contributions

WT, SJ, KY, and LD designed the experiments. WT, DV, JC, and WB performed the experiments. WT, DV, JC, WB, DK, and BS analyzed the data. WT, DV, BS, SJ, KY, and LD wrote and revised the manuscript.

## Conflict of Interest Statement

The authors declare that the research was conducted in the absence of any commercial or financial relationships that could be construed as a potential conflict of interest.
